# Boys Who Smoke

**Published:** 1868-12

**Authors:** 


Boys who Smoke.—Dr. Decaisne in the course of investigations on the influence of tobacco on the circulation, has been struck with the large number of boys, aged from nine to fifteen years, who smoke; and has been led to inquire into the connection of this habit with impairment of the general health. He has observed 38 boys, aged from 9 to 15, who smoked more or less. Of these, distinct symptoms were present in 27. In 22, there were various disorders of the circulation—*bruit de souffle* in the neck, palpitation, disorders of digestion, slowness of intellect, and a more or less marked taste for strong drink. In 3, the pulse was intermittent. In 8, there was found on examination more or less marked diminution of the red corpuscles; in 12, there was rather frequent epistaxis; 10 had disturbed sleep; and 4 had slight ulcerations of the mucous membrane of the mouth, which disappeared on ceasing from the use of tobacco for some days. In children who are very well nourished, the disorder was, in general less marked. As to the ages, 8 of the boys were from 9 to 12 years old; 19 from 12 to 15. The duration of the habit of smoking was: in 11, from six months to a year; and in 16, more than two years. The ordinary treatment of anæmia in general produced no effect as long as the smoking was continued; but when this was desisted from, health was soon perfectly restored, if there were no organic disease.—*Brit. Med. Journal*, Sept. 26, 1868.

